# The Adaptive Characteristics of Cholesterol and Bile Acid Metabolism in Nile Tilapia Fed a High-Fat Diet

**DOI:** 10.1155/2022/8016616

**Published:** 2022-11-24

**Authors:** Rui-Xin Li, Yi-Fan Qian, Wen-Hao Zhou, Jun-Xian Wang, Yan-Yu Zhang, Yuan Luo, Fang Qiao, Li-Qiao Chen, Mei-Ling Zhang, Zhen-Yu Du

**Affiliations:** LANEH, School of Life Sciences, East China Normal University, Shanghai, China

## Abstract

Since high-fat diet (HFD) intake elevates liver cholesterol and enhanced cholesterol-bile acid flux alleviates its lipid deposition, we assumed that the promoted cholesterol-bile acid flux is an adaptive metabolism in fish when fed an HFD. The present study investigated the characteristic of cholesterol and fatty acid metabolism in Nile tilapia (*Oreochromis niloticus*) after feeding an HFD (13% lipid level) for four and eight weeks. Visually healthy Nile tilapia fingerlings (average weight 3.50 ± 0.05 g) were randomly distributed into four treatments (4-week control diet or HFD and 8-week control diet or HFD). The liver lipid deposition and health statue, cholesterol/bile acid, and fatty acid metabolism were analyzed in fish after short-term and long-term HFD intake. The results showed that 4-week HFD feeding did not change serum alanine transaminase (ALT) and aspartate transferase (AST) enzyme activities, along with comparable liver malondialdehyde (MDA) content. But higher serum ALT and AST enzyme activities and liver MDA content were observed in fish fed 8-week HFD. Intriguingly, remarkably accumulated total cholesterol (mainly cholesterol ester, CE) was observed in the liver of fish fed 4-week HFD, along with slightly elevated free fatty acids (FFAs) and comparable TG contents. Further molecular analysis in the liver showed that obvious accumulation of CE and total bile acids (TBAs) in fish fed 4-week HFD was mainly attributed to the enhancement of cholesterol synthesis, esterification, and bile acid synthesis. Furthermore, the increased protein expressions of acyl-CoA oxidase 1/2 (Acox1 and Acox2), which serve as peroxisomal fatty acid *β*-oxidation (FAO) rate-limiting enzymes and play key roles in the transformation of cholesterol into bile acids, were found in fish after 4-week HFD intake. Notably, 8-week HFD intake remarkably elevated FFA content (about 1.7-fold increase), and unaltered TBAs were found in fish liver, accompanied by suppressed Acox2 protein level and cholesterol/bile acid synthesis. Therefore, the robust cholesterol-bile acid flux serves as an adaptive metabolism in Nile tilapia when fed a short-term HFD and is possibly via stimulating peroxisomal FAO. This finding enlightens our understanding on the adaptive characteristics of cholesterol metabolism in fish fed an HFD and provides a new possible treatment strategy against metabolic disease induced by HFD in aquatic animals.

## 1. Introduction

Currently, to obtain the protein-sparing effect, high-fat diets (HFD) have been extensively formulated to meet the energy demand of fish growth. However, HFD usually leads to severe accumulation of lipids, mainly triglyceride (TG), in fish [1, 2]. Also, the elevation of cholesterol is a common phenomenon in fish body. In fact, cholesterol is an essential lipid involved in many biological processes for providing a precursor for the biosynthesis of bile acids, vitamin D, and steroid hormones in animals. However, excessive free cholesterol (FC) is the major risk factor for nonalcoholic fatty liver disease (NAFLD) [3–5]. One of the mechanisms by which FC causes lipotoxicity is the generation of reactive oxygen species production and mitochondrial damage [6, 7]. In fish, limited studies focus on the mechanism linking HFD-induced obesity to elevated cholesterol in the liver, while the physiological role of cholesterol in HFD-fed fish is not fully understood.

There is a strong link between cholesterol and fatty acids as previous studies reported that free fatty acids (FFAs) stimulate hepatic cholesterol biosynthesis [8, 9]. It is well-known that cholesterol biosynthesis is a complex enzymatic biochemical process that initializes with substrate acetyl coenzyme A (acetyl-CoA). The acetyl-CoA for cholesterol biosynthesis mainly derives from the following two sources: one is generated by citrate cleavage, and the other is from acetate produced by fatty acid *β*-oxidation (FAO) [10, 11]. A recent study about the mechanism of diabetes-induced hypercholesterolemia in mice revealed that stimulated liver peroxisomal FAO generated considerable free acetate, a precursor for cholesterol biosynthesis [12]. In a normal physiological condition, cholesterol homeostasis is tightly regulated in animals based on the cellular cholesterol level [13, 14], and high cholesterol levels always promote cholesterol esterification and elimination to prevent lipotoxicity [15, 16]. Previous reports in obese mice demonstrated that the enhancement of cholesterol metabolism, including cholesterol and bile acid efflux, alleviated the deposition of lipids such as cholesterol, FFA, and TG in the liver [17–19]. Therefore, to some extent, enhancing the conversion of FFA to cholesterol and bile acids is an important metabolism pathway for reducing FFA accumulation in the liver.

Previous researches believed that fish could grow normally without exogenous dietary cholesterol supplementation [20, 21], indicating that robust regulation of cholesterol metabolism exists in fish and is enough to meet body cholesterol requirements compared with mammals. Additionally, although HFD feeding would disturb lipid homeostasis, resulting in excessive lipid accumulation and severe lipotoxicity in fish, there is a protective mechanism against the lipotoxicity of FFA in response to temporary HFD intake [22]. However, such a protective mechanism has not been well illustrated in fish. We assumed that its robust regulation of cholesterol metabolism is an efficient process to alleviate HFD-induced lipotoxicity. Therefore, further studies are required to demonstrate the physiological role of cholesterol in HFD-fed fish. Such knowledge helps to give insight into the role of cholesterol metabolism on metabolic disorders and provides a new possible treatment strategy against metabolic disease in aquatic animals. The present study mainly investigated the changes in cholesterol and fatty acid metabolism after short-term (4 weeks) or long-term (8 weeks) HFD feeding in Nile tilapia (*Oreochromis niloticus*), which is a global farmed fish widely used in metabolic studies [22, 23]. Previous studies in Nile tilapia showed that approximately 6% lipid level was the optimal dietary lipid level [24–26], whereas 13% lipid level showed a negative impact on growth rates and feed utilization [27]. After the feeding trials, organic index, biochemical indicators, and gene/protein expression involved in cholesterol and fatty acid metabolism were analyzed.

## 2. Materials and Methods

### 2.1. Experimental Animals and Diet and Sample Collection

The animal study was conducted strictly conforming to the procedures approved by the Committee on Ethics of Animal Experiments of East China Normal University (approval number F20190101). Nile tilapia were acclimated into tanks (water volume at 200 liters each tank within an indoor circulating water system) and fed a commercial diet (including 35% protein and 5% lipid, Tongwei, Co. Ltd., Chengdu, China) twice daily (09:00 h and 17:00 h) for two weeks. After acclimatization, visually healthy Nile tilapia fingerlings (average weight 3.50 ± 0.05 g) were randomly distributed into tanks (30 fish in each tank, three replicates, and four treatments). The experimental diets were formulated to contain two different lipid levels (6% and 13%; control diet and HFD, respectively), along with approximately 39% protein and 32% carbohydrate levels according to previous studies [24–27]. All dry ingredients were finely ground through 60-mesh screen and thoroughly mixed, and then, soybean oil and distilled water were added to thoroughly blend for producing 2 mm diameter pellets with a twin-screw extruder (South China University of Technology, Guangzhou, China). All the experimental diets were air-dried and stored at −20°C until used. The fish were fed with control diet and HFD (see Table 1) twice daily (09:00 h and 17:00 h) at 4% of their body weight per day for four weeks or eight weeks. The weight of fish in each tank was recorded to adjust diet intake each week. During the whole feeding trial, water temperature, total ammonia-nitrogen, and dissolved oxygen ranged from 27.00 to 29.00°C, 0 to 0.20 mg/L, and 5.00 to 6.50 mg/L, respectively.

At the end of the 4-week or 8-week feeding trial, all fish of control and HFD (three tanks from each treatment) were deprived of food overnight, then anesthetized with MS-222 (0.1 g/L, Sigma, USA), counted, and bulk weighed to analyze the survival rate and growth performance. Four fish were randomly collected from each tank and individually weighed and measured to calculate the condition factor (CF). Then, blood samples were collected from the fish caudal vein using 1 mL syringes. Serum samples were obtained for biochemical analyses after the centrifugation at 3000 rpm for 10 min at 4°C. Additionally, the fish were dissected to obtain mesenteric fat and liver tissues. The weights of these tissues were noted to determine the mesenteric fat index (MFI) and hepatosomatic index (HSI). Serum, liver, and intestinal content samples were immediately frozen into liquid nitrogen and stored at −80°C until further analysis.

### 2.2. Biochemical and Histological Analyses

The total lipid in the liver was extracted by using chloroform/methanol (2 : 1, *v*/*v*) according to previously described [28]. Liver tissue and intestinal content were homogenized in phosphate-buffered saline (PBS) at a ratio of 1 : 10 (*w* : *v*) and then centrifuged (4°C, 3000 rpm, 10 min). The supernatants were collected and used for biochemical assays. The total cholesterol (TC) and free cholesterol (FC) were determined using specific commercial assay kits (Solarbio, Beijing, China) according to the method of enzymatic assays [29]. Briefly, the esterified cholesterol was hydrolyzed to free cholesterol by cholesterol esterase; then, the free cholesterol was oxidized to *Δ*4-cholesterone and H_2_O_2_ by cholesterol oxidase. Finally, the H_2_O_2_ was oxidized by peroxidase in the presence of 4-aminoantipyrine and phenol to form red quinones, which could be measured at 500 nm by using spectrophotometers. TG, free fatty acids (FFAs), total bile acids (TBAs), low-density lipoprotein cholesterol (LDL-C), and high-density lipoprotein cholesterol (HDL-C) in serum, liver, or intestinal content were measured using specific commercial assay kits (Nanjing Jiancheng Bioengineering Institute, Nanjing, China) according to the manufacturer's instructions. Briefly, TG was measured with glycerol phosphate oxidase/peroxidase and hydrogen peroxide (GPO/PAP) method. FFA was detected by acyl CoA synthetase and acyl CoA oxidase (ACS-ACOD) method. TBA was detected in the presence of Thio-NAD and 3-*α*-hydroxysteroid dehydrogenase (3-*α*-HSD), which converted bile acids to 3-keto steroids and Thio-NADH. LDL-C and HDL-C were determined with cholesterol esterase and cholesterol oxidase after being selectively solubilized by different specific surfactants (see instructions for details). Very low-density lipoprotein cholesterol (VLDL-C) was assessed by ELISA (enzyme-linked immunosorbent assay) kit (Hengyuan Biotech Co., China) according to the manufacturer's protocols. The activities of aspartate transferase (AST) and alanine transaminase (ALT) were detected following the method [30]. Briefly, AST catalyzed *α*-ketoglutaric acid and aspartate to produce glutamic acid and oxaloacetic acid. Oxaloacetic acid was further decarboxylated to form pyruvate, which react with 2,4-dinitrophenylhydrazine to produce 2,4-dinitrophenylhydrazone. ALT facilitated the conversion of alanine and *α*-ketoglutarate to glutamate and pyruvate, which reacted with 2,4-dinitrophenylhydrazine to form a yellow pyruvate phenylhydrazone. Superoxide dismutase (SOD) activity and malondialdehyde (MDA) content were assessed according to the methods described previously [31, 32]. SOD catalyzed the dismutation of the superoxide anion into hydrogen peroxide and O_2_, and superoxide anions acted on WST-1 to produce a water-soluble formazan dye. MDA reacted with thiobarbituric acid (TBA) to generate an MDA-TBA adduct, which could be quantified colorimetrically. Liver tissues were fixed in 4% paraformaldehyde solution and processed for histology in paraffin wax and then cut into 5 *μ*m thick sections for neutral lipid staining following the procedure [33].

### 2.3. Quantitative Real-Time PCR

The quantitative real-time PCR was conducted according to our previous study [34]. Total RNA was extracted from the liver using TRIzol reagent (Takara, Dalian, China) according to the manufacturer's protocol. Briefly, liver tissues (about 30 mg) were homogenized and centrifuged in lysis buffer. The supernatant was mixed with isopropanol for the retention of RNA. After the sediment (RNA) was washed appropriately, it was eluted in 50 *μ*L of RNAse-free H_2_O. The concentrations of the total RNA obtained were measured using NanoDrop 2000 Spectrophotometer. Next, cDNA was obtained by using the HiScript III RT SuperMix for qPCR (with gDNase, Vazyme Biotech Co., Ltd., Nanjing, China) according to the manufacturer's protocols. Briefly, after eliminating genomic DNA (gDNA) with DNase, the cDNA was synthesized in the presence of HiScript III RT SuperMix. The reaction mixture was incubated under the following condition: 37°C for 15 min, 85°C for 5 s, and 4°C forever. Finally, the qPCR was performed in the CFX96 Real-Time RCR system (Bio-Rad, CA) with ChamQ Universal SYBR qPCR Master Mix (Vazyme Biotech Co., Ltd., Nanjing, China) following the manufacturer's instructions. The region of interest (ROI), background, and pure dye spectra of qPCR instrument were regularly calibrated according to the instrument's manual. The amplification condition applied to qPCR was as follows: 95°C for 3 min, followed by 40 cycles of 95°C for 10 s and 60°C for 30 s. The housekeeping (*β*-actin and elongation factor 1 alpha (EF1*α*), both of which stably expressed in different dietary groups) and target gene primers used for qPCR are present in Table 2. The amplification efficiencies of the candidate genes ranged from 90 to 105%. The expression levels of target genes were determined by using the 2^-∆∆Ct^ method [35].

### 2.4. Western Blotting

The Western blotting was performed following the method described previously [36]. Liver total proteins were obtained by using the ice-cold RIPA lysis buffer supplemented with protease inhibitors (Beyotime Biotechnology, China), and their concentrations were determined following the protocol of total protein quantification assay (New Cell & Molecular Biotech, China). The supernatant protein was mixed with 5× SDS loading buffer and boiled for 15 min. The obtained protein samples were resolved by sodium dodecyl sulfate-polyacrylamide gel electrophoresis (SDS-PAGE) and transferred onto nitrocellulose membranes. Immunoblots were further incubated with 5% BSA in phosphate-buffered saline with Tween 20 (TBST) buffer. The target proteins of membrane were incubated with primary antibodies (anti-ACAT1, 1 : 800, A13273, ABclonal; anti-ACAT2, 1 : 1000, A1399, ABclonal; anti-CYP7A1, 1 : 800, A10615, ABclonal; anti-CPT1a, 1 : 800, 15184-1-AP, Proteintech; anti-ACLY, 1 : 800, ab40793, Abcam; ACOX1, 1 : 800, 10957-1-AP, Proteintech; ACOX2, 1 : 800, A12796, ABclonal; anti-*α*-Tubulin, 1 : 1000, M-1051-1, Huabio; and anti-*β*-ACTIN, AB0035, Abways) overnight at 4°C. After washing in TBST, bolts were incubated with secondary antibodies for 1 h at room temperature. The protein images were visualized by using an Odyssey CLx Imager (Licor, USA).

### 2.5. Statistical Analyses

The obtained data were analyzed by two-way ANOVA after testing the normality and homogeneity of variances using the Shapiro-Wilk test and Levene's test, respectively. Significant differences (*P* ≤ 0.05) were determined by Tukey's HSD post hoc test. Asterisks indicate the differences between the C and HFD groups in the same feeding period. Hash symbols indicate the differences between the 4-week and 8-week feeding trials of the same lipid level diet. All the obtained experimental data were analyzed by using the SPSS Statistics 23.0 software (IBM, Michigan Avenue, USA). All values in the results are presented as the means ± SD (standard deviation).

## 3. Results

### 3.1. HFD Feeding Caused Hyperlipidemia

**Figure 1:**
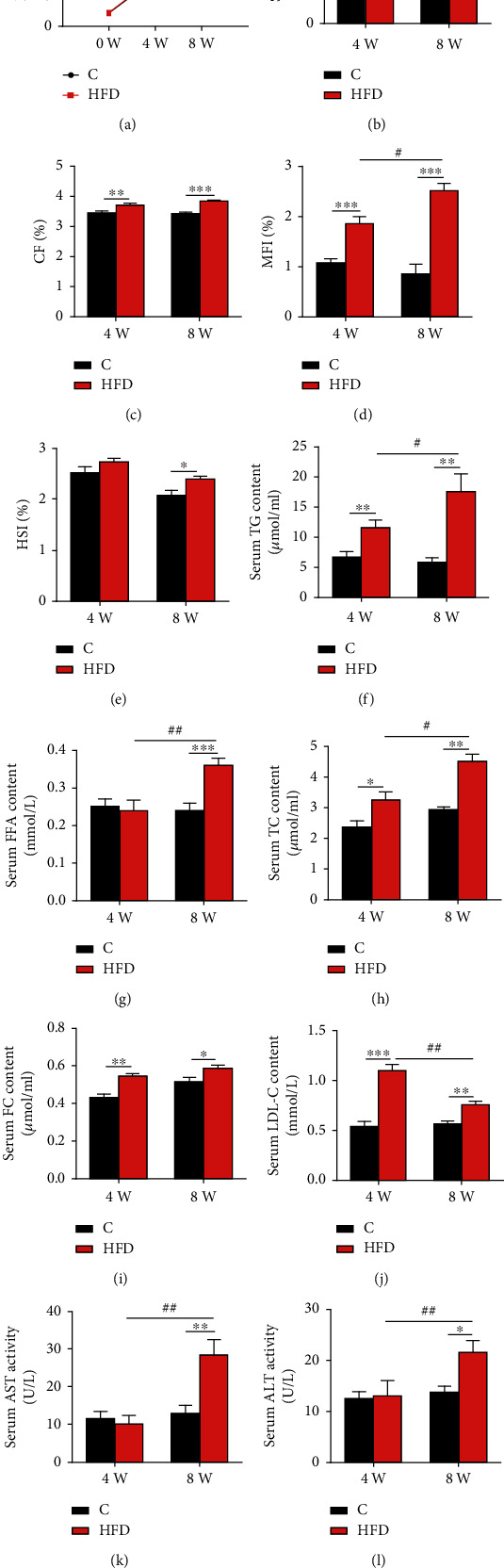
Effects of HFD on growth performance, organ indices, serum lipid profiles, and liver health in Nile tilapia after 4-week and 8-week feeding trials. (a) Body weight (*n* = 3 tanks, 30 fish/tank); (b) survival rate (SR, *n* = 3 tanks); (c) condition factor (CF, *n* = 12); (d) mesenteric fat index (MFI, *n* = 12); (e) hepatosomatic index (HSI, *n* = 12); (f, g) triglyceride (TG) and free fatty acid (FFA) in serum (*n* = 9); (h, i) total cholesterol (TC) and free cholesterol (FC) in serum (*n* = 9); (j) low-density lipoprotein cholesterol (LDL-C) in serum (*n* = 9); (k, l) serum aspartate aminotransferase (AST) and alanine aminotransferase (ALT) enzyme activities (*n* = 9). Data are represented as means ± SD; asterisks indicate the differences between the C and HFD groups in the same feeding trial, ^∗^*P* < 0.05, ^∗∗^*P* < 0.01, ^∗∗∗^*P* < 0.001; hash symbols indicate the differences between 4-week and 8-week feeding trials of the same lipid level diet, ^#^*P* < 0.05, ^##^*P* < 0.01, ^###^*P* < 0.001; C: control diet; HFD: high-fat diet. SR (%) = final number of fish × 100/initial number of fish. CF (%) = body weight × 100/body length^3^. MFI (%) = 100 × mesenteric fat weight/individual fish body weight. HSI (%) = 100 × liver weight/individual fish body weight.

HFD intake (4 or 8 weeks) did not affect survival rate of fish (Figure 1(b)), but after the 4-week HFD feeding trial, although no significant difference in body weight was found between the control and HFD groups (Figure 1(a)), higher CF and MFI were observed in the HFD-fed Nile tilapia (Figures 1(c) and 1(d)). However, 8 weeks of HFD intake significantly increased the body weight of fish (Figure 1(a)) and caused remarkably higher CF, MFI, and HSI than the control (Figures 1(c)–1(e)). Furthermore, 4-week HFD intake significantly elevated serum TG (Figure 1(f)), TC (Figure 1(h)), and FC (Figure 1(i)) levels, which further accumulated after 8 weeks of HFD feeding. Notably, 4-week HFD feeding neither changed serum FFA level (Figure 1(g)) nor serum ALT and AST enzyme activities (Figures 1(k) and 1(l)), two important indicators of liver function. But higher serum FFA levels (Figure 1(g)), along with elevated ALT and AST (Figures 1(k) and 1(l)) enzyme activities were observed in the 8-week HFD-fed fish. Additionally, the 4-week HFD feeding elevated serum LDL-C content, which conversely diminished in fish after 8-week HFD intake (Figure 1(j)). Generally, short-term (4 weeks) HFD intake caused hyperlipidemia that was aggravated after long-term (8 weeks) HFD feeding and is accompanied by impaired liver functions in fish.

### 3.2. Biochemical Changes in Lipid Metabolism in the Fish Fed HFD

**Figure 2:**
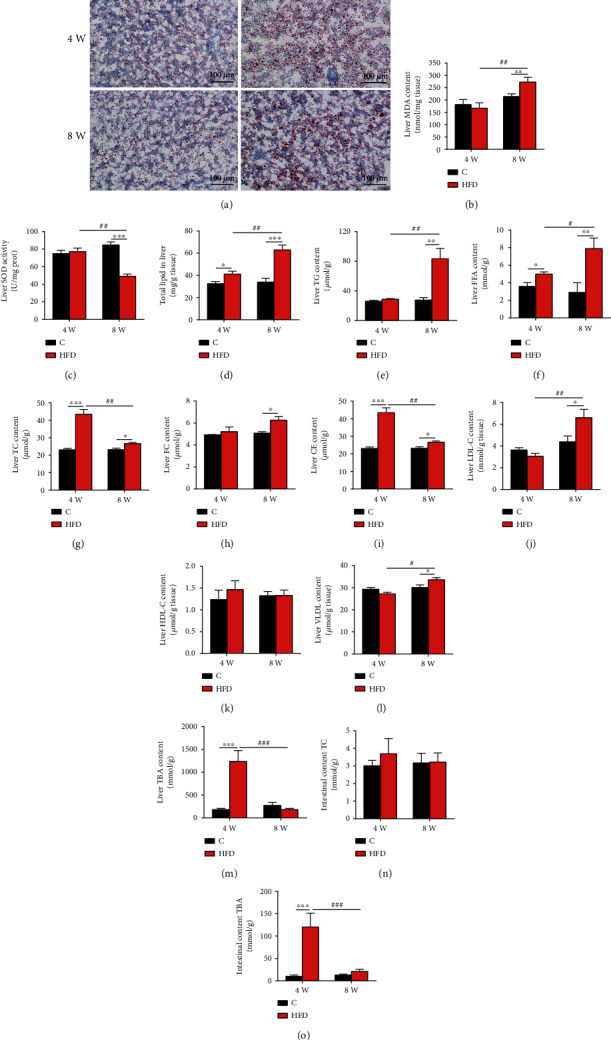
Effects of HFD on liver lipid profile and total bile acids in the liver and intestinal content of Nile tilapia after 4-week and 8-week feeding trials. (a) Oil red O staining of liver, scale bars: 100 *μ*m (*n* = 4); (b) liver malondialdehyde (MDA) content (*n* = 9); (c) liver superoxide dismutase (SOD) enzyme activity (*n* = 9); (d) liver lipid content (*n* = 9); (e, f) liver triglyceride (TG) content and free fatty acid (FFA) contents (*n* = 9); (g–i) liver total cholesterol (TC), free cholesterol (FC), and cholesterol ester (CE) contents (*n* = 9); (j–l) liver low-density lipoprotein cholesterol (LDL-C), high-density lipoprotein cholesterol (HDL-C), and very-low-density lipoprotein (VLDL) contents (*n* = 9); (m) liver total bile acid (TBA) content (*n* = 9); (n, o) TC and TBA contents in intestinal content (*n* = 9). Data are represented as means ± SD; asterisks indicate the differences between the C and HFD groups in the same feeding trial, ^∗^*P* < 0.05, ^∗∗^*P* < 0.01, ^∗∗∗^*P* < 0.001; hash symbols indicate the differences between 4-week and 8-week feeding trials of the same lipid level diet, ^#^*P* < 0.05, ^##^*P* < 0.01, ^###^*P* < 0.001; C: control diet; HFD: high-fat diet.

Furthermore, oil red O staining and biochemical analysis showed that lipid accumulation was evident in the liver of fish fed an HFD for 4 weeks and became more severe in fish fed 8-week HFD (Figures 2(a) and 2(d)). Accordingly, although 4-week HFD intake has no effect on liver MDA content and SOD enzyme activity in fish (Figures 2(b) and 2(c)), increased liver MDA content and decreased SOD enzyme activity were found in the fish after 8-week HFD feeding. Moreover, significantly elevated FFA (Figure 2(f)) rather than TG (Figure 2(e)) contents were observed in the liver of fish fed 4-week HFD. However, remarkably accumulated TC (increased by onefold) was found in fish liver (Figure 2(g)). The increased TC was mainly CE (Figure 2(i)) while FC elevated slightly (Figure 2(h)). In addition, liver LDL-C, HDL-C, and VLDL contents were not changed in fish after the 4-week feeding trial (Figures 2(j)–2(l)). However, liver lipid accumulation caused by 8-week HFD intake was mainly reflected in elevated TG (increased by about 1.4-fold, Figure 2(e)) and considerably accumulated FFA (increased by about 1.5-fold, Figure 2(f)). In addition, there was significant accumulation of TC (Figure 2(g)), which was mainly in FC (Figure 2(h)) rather than CE (Figure 2(i)) in the liver. Liver LDL-C and VLDL contents were significantly increased in HFD-fed fish for 8 weeks (Figures 2(j) and 2(l)). Furthermore, although no significantly altered TC content was detected in the intestinal content of fish fed HFD (Figure 2(n)), the fish exhibited remarkably increased TBA content in the liver and intestinal content after 4-week feeding trial (Figures 2(m) and 2(o)). However, after 8-week feeding trial, both of these parameters were unchanged in fish between the control and HFD groups (Figures 2(m) and 2(o)). These results indicated that 4-week HFD intake caused a high accumulation of CE rather than TG in fish liver, but elevated TG and FFA contents primarily accounted for the liver lipid deposition of fish fed 8-week HFD.

### 3.3. Molecular Changes in Cholesterol Metabolism in the Fish Fed HFD

**Figure 3:**
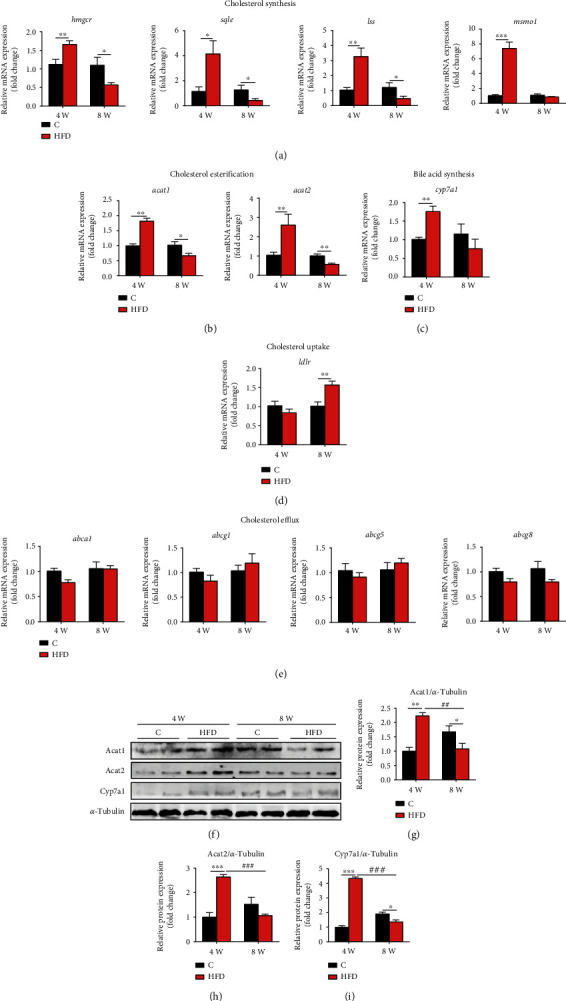
Effects of HFD on cholesterol and bile acid metabolism in the liver of Nile tilapia after 4-week and 8-week feeding trials. (a–e) mRNA expression of genes related to cholesterol synthesis (a), esterification (b), uptake (d), efflux (e), and bile acid synthesis (c) in the liver (*n* = 9); (f–i) relative expression of proteins related to cholesterol esterification (g, h) and bile acid synthesis (i) in the liver (*n* = 4). Data are represented as means ± SD; asterisks indicate the differences between the C and HFD groups in the same feeding trial, ^∗^*P* < 0.05, ^∗∗^*P* < 0.01, ^∗∗∗^*P* < 0.001; hash symbols indicate the differences between 4-week and 8-week feeding trials of the same lipid level diet, ^#^*P* < 0.05, ^##^*P* < 0.01, ^###^*P* < 0.001; C: control diet; HFD: high-fat diet. *hmgcr*: 3-hydroxy-3-methylglutaryl-coenzyme A reductase; *sqle*: squalene monooxygenase; *lss*: lanosterol synthase; *msmo1*: methylsterol monooxygenase 1; *acat1*: acetyl-CoA acetyltransferase 1; *acat2*: acetyl-CoA acetyltransferase 2; *cyp7a1*: cholesterol 7alpha-hydroxylase; *ldlr*: low-density lipoprotein receptor; *abca1*: ATP binding cassette subfamily A member 1; *abcg1*: ATP binding cassette subfamily G member 1; *abcg5*: ATP binding cassette subfamily G member 5; *abcg8*: ATP binding cassette subfamily G member 8.

Further molecular analysis in the liver showed that 4-week HFD intake markedly upregulated the expression of genes associated with cholesterol synthesis (3-hydroxy-3-methylglutaryl-coenzyme A reductase, *hmgcr*; squalene monooxygenase, *sqle*; lanosterol synthase, *lss*; methylsterol monooxygenase 1, *msmol*; Figure 3(a)), cholesterol esterification (acetyl-CoA acetyltransferase 1, *acat1*; acetyl-CoA acetyltransferase 2, *acat2*; Figure 3(b)), and bile acid synthesis (cholesterol 7alpha-hydroxylase, *cyp7a1*; Figure 3(c)) but did not affect gene expressions related to cholesterol uptake (low-density lipoprotein receptor, *ldlr*; Figure 3(d)) and efflux (ATP binding cassette subfamily A member 1, *abca1*; *abcg1*, ATP binding cassette subfamily G member 1; ATP binding cassette subfamily G member 5, *abcg5*; ATP binding cassette subfamily G member 8, *abcg8*; Figure 3(e)). Accordingly, protein expression of Acat1 (Figure 3(g)), Acat2 (Figure 3(h)), and Cyp7a1 (Figure 3(i)) was significantly upregulated in the liver of fish after 4-week HFD feeding (Figure 3(f)). However, 8-week HFD feeding significantly inhibited the expression of genes related to cholesterol synthesis (*hmgcr*, *sqle*, and *lss*; Figure 3(a)) and protein related to cholesterol esterification (Acat1 and Acat2; Figures 3(g) and 3(h)). In addition, this long-term HFD feeding did not affect the expression of genes related to cholesterol efflux (*abca1*, *abcg1*, *abcg5*, and *abcg8*; Figure 3(e)) and bile acid synthesis (*cyp7a1*; Figure 3(c)) but enhanced cholesterol uptake-related gene *ldlr* (Figure 3(d)). These results indicated that the evident accumulation of cholesterol and bile acids in the liver of fish fed 4-week HFD was mainly due to the enhancement of cholesterol synthesis, esterification, and bile acid synthesis. Eight-week HFD intake-induced cholesterol accumulation was linked to the suppressed hepatic LDL-C uptake.

### 3.4. Molecular Changes in TG and FA Metabolism in the Fish Fed HFD

**Figure 4:**
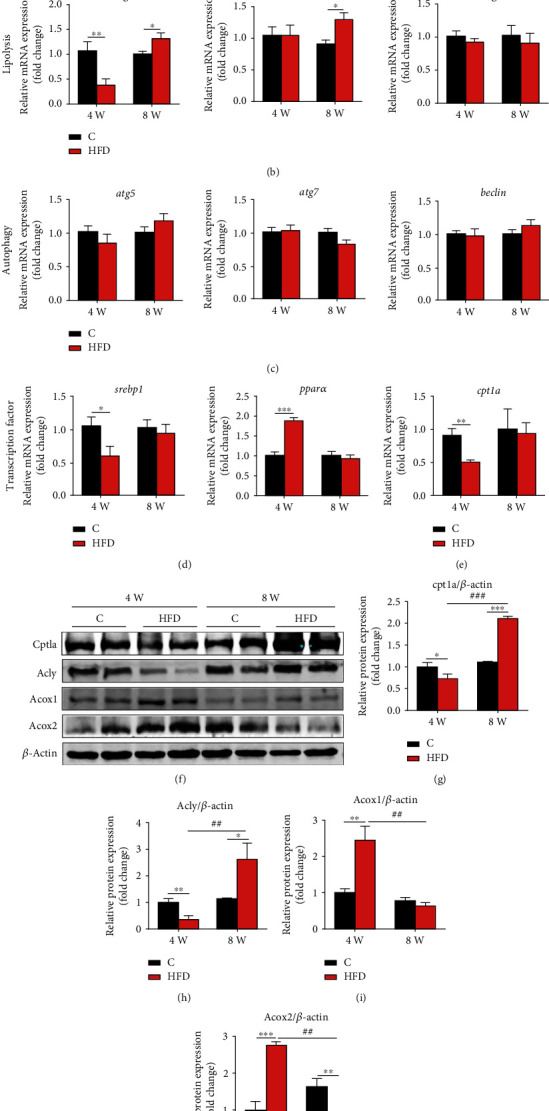
Effects of HFD on fatty acid synthesis, lipolysis, autophagy, and fatty acid *β*-oxidation in the liver of Nile tilapia after 4-week and 8-week feeding trials. (a–c) mRNA expression of genes related to FA synthesis (a), lipolysis (b), and autophagy (c) in the liver (*n* = 9); (d, e) mRNA expression of genes related to lipid metabolism transcription factors (d) and mitochondrial FAO (e, *n* = 9); (f–j) relative expression of proteins related to mitochondrial (g) and peroxisomal (i, j) FAO and cytosolic acetyl-CoA synthesis (h) in the liver (*n* = 4). Data are represented as means ± SD; asterisks indicate the differences between the C and HFD groups in the same feeding trial, ^∗^*P* < 0.05, ^∗∗^*P* < 0.01, ^∗∗∗^*P* < 0.001; hash symbols indicate the differences between 4-week and 8-week feeding trials of the same lipid level diet, ^#^*P* < 0.05, ^##^*P* < 0.01, ^###^*P* < 0.001; C: control diet; HFD: high-fat diet. *fasn*: fatty acid synthase; *acly*: ATP citrate lyase; *accα*: acetyl-CoA carboxylase alpha; *atgl*: adipose triglyceride lipase; *hsl*: hormone-sensitive lipase; *mgl*: monoacylglycerol lipase; *atg5*: autophagy-related gene 5; *atg7*: autophagy-related gene 7; *srebp1*: sterol regulatory element binding protein 1; *pparα*: peroxisome proliferator-activated receptor *α*; *cpt1a*: carnitine palmitoyl transferase 1a; Acox1/2: acyl-CoA oxidase 1/2.

Because cholesterol metabolism is closely related to fatty acid, we further performed the molecular analysis on fatty acid metabolism. The results showed that 4-week HFD intake significantly inhibited the gene expressions associated with fatty acid synthesis (*fasn*, fatty acid synthase; *acly*, ATP citrate lyase; *accɑ*, acetyl-CoA carboxylase alpha; Figure 3(a)) and lipolysis (*atgl*, adipose triglyceride lipase; Figure 4(b)) but did not alter the expression of genes related to autophagy (*atg5*/*7*, autophagy-related gene 5/7 and *beclin*; Figure 4(c)). Interestingly, peroxisome proliferator-activated receptor *α* (*pparα*, a key transcription factor of fatty acid *β*-oxidation; Figure 4(d)) and Acox1/2 (acyl-CoA oxidase 1/2; peroxisomal *β*-oxidation rate-limiting enzymes; Figures 4(f), 4(i), and 4(j)) were significantly upregulated in gene and/or protein levels, but Cpt1a (carnitine palmitoyl transferase 1a, a mitochondrial *β*-oxidation key transporter protein; Figures 4(f) and 4(g)) and Acly (Figures 4(f) and 4(h)) protein expressions were significantly inhibited in the liver of fish fed 4-week HFD. However, 8-week HFD intake significantly stimulated the gene expressions related to lipolysis (*atgl* and *hsl*; Figure 4(b)) and suppressed the peroxisomal *β*-oxidation (Acox1/2; Figures 4(i) and 4(j)), but it did not affect fatty acid synthesis (*fasn*, *acly*, *accα*, and *srebp1*; Figures 4(a) and 4(d)), autophagy (*atg5*/*7* and *beclin*; Figures 4(c)), and mitochondrial *β*-oxidation (Cpt1a; Figures 4(e) and 4(g)). These results suggested that liver peroxisomal *β*-oxidation was activated after 4-week HFD feeding but was inhibited when fed HFD for 8 weeks.

## 4. Discussion

### 4.1. Short-Term HFD Feeding Shows an Adaptive Mechanism against Lipotoxicity in the Liver

It is well-known that HFD leads to lipid accumulation in most animals. In fish, obviously increased mass of visceral adipose tissue, elevated plasma TG, FFA, cholesterol levels, and liver lipid content were observed after HFD intake [37, 38]. It should be pointed out that in response to short-term HFD intake, high levels of circulating FFAs are primarily stored in the form of TG in the adipose tissue of fish. This process has been identified as an adaptive mechanism to ease the FFA-induced lipotoxicity in animals [39]. For instance, our previous study in Nile tilapia reported that the increased number of adipocytes is the primary strategy to cope with HFD intake at the early stages of the progression of fatty liver, along with relatively stable TG content in the liver [22]. Consistently, higher CF and MFI but not HSI were also observed in the fish fed with HFD for 4 weeks in the present study. Although hyperlipidemia occurred in fish after 4 weeks of HFD intake, there is only a slight elevation of FFA in the fish liver while the TG content remains stable. The liver is the central metabolic organ, where dysregulation of lipid metabolism is a critical factor in liver steatosis [40]. In the present study, 4-week HFD intake significantly inhibited fatty acid synthesis (*fasn*, *acly*, and *srebp1*) and lipolysis (*atgl*) processes. These results might partially explain why short-term HFD feeding did not cause liver damage, as evident by comparable serum ALT and AST enzyme activities, liver MDA content, and liver SOD enzyme activity in fish. We proposed that liver FFA level was tightly controlled within normal fluctuation levels so that the liver would not be impaired in fish, which supported the protective mechanism against lipotoxicity in the liver of fish after short-term HFD intake.

### 4.2. Short-Term HFD Feeding Triggers Cholesterol-Bile Acid Flux to Eliminate FFA-Derived Cholesterol

Cholesterol biosynthesis begins with acetyl-CoA, which could be supplied by FAO, a critical lipid catabolism process involved in the degradation of FFA [10, 11]. This might explain the previous finding that there is a strong link between cholesterol and FFA [8, 9]. In the present study, short-term HFD feeding caused cholesterol accumulation (mainly CE) rather than TG in fish liver, accompanied by enhanced cholesterol synthesis (*hmgcr*, *sqle*, *lss*, and *msmo1*) and esterification (Acat1 and Acat2). The obvious elevation of CE along with slightly increased FC content might be explained by the fact that FC, as a polar lipid with lipotoxicity, can be efficiently converted to CE by ACATs, and then, CE is subsequently stored in lipid droplets [16]. Also, the liver eliminates the excessive cholesterol within cells mainly via the transformation of cholesterol to bile acids, which are then being excreted into the gallbladder and intestine [41, 42]. Bile acid synthesis exclusively occurs in the liver and is the only quantitatively significant cholesterol catabolic mechanism [41, 42]. In the present study, short-term HFD feeding increased TBA content in the liver and intestinal content, along with promoted bile acid synthesis (such as Cyp7a1, the first rate-limiting enzyme) in fish liver. These results indicated that the enhanced bile acid synthesis and excretion processes also served as a protective mechanism for eliminating FFA-derived cholesterol in the liver of fish after short-term HFD intake.

Of note, the gross energy is different between the control and high-fat diets in the present study. Thus, it raises an interesting question that whether the adaptive metabolic characteristics of cholesterol and bile acid also exist in Nile tilapia fed a high-energy diet. In fact, previous study showed that the high-carbohydrate diet intake also led to high level of hepatic cholesterol and bile acids [43], indicating the robust cholesterol and bile acid metabolism in the liver of fish under the high carbohydrate nutrition. Therefore, we hypothesized that fish could alleviate the metabolic disorders caused by the high-energy diet through enhancing cholesterol and bile acid metabolism. Future studies are necessary to assess the variety and content of bile acids in the gallbladder of fish and reveal their physiological functions in fish under the high energy nutrition.

### 4.3. Short-Term HFD Feeding Enhanced Peroxisomal FAO-Mediated Cholesterol and Bile Acid Synthesis

**Figure 5:**
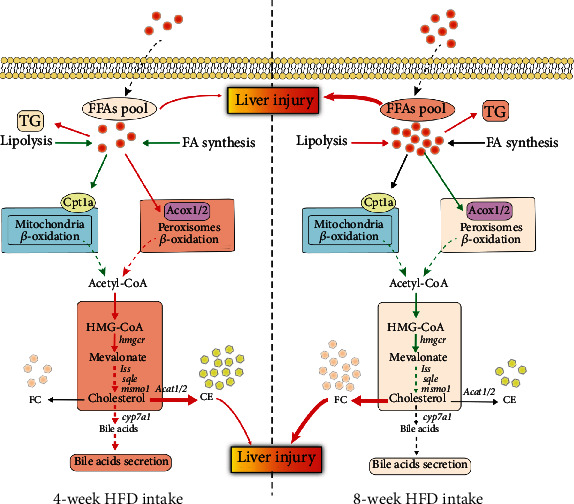
A hypothetical diagram showing that short-term (4 weeks) HFD intake triggers protective mechanism against FFA accumulation and liver injury through promoting hepatic cholesterol/bile acid synthesis and flux, which might be linked to stimulated peroxisomal *β*-oxidation for supply of substrate acetyl-CoA. Deficient hepatic peroxisomal *β*-oxidation might block the conversion of FFAs to cholesterol and bile acids and then failed to alleviate FFA accumulation in fish after long-term (8 weeks) HFD intake. Therefore, peroxisomal *β*-oxidation-mediated cholesterol-bile acid flux might serve an adaptive metabolism in Nile tilapia fed an HFD. FFAs: free fatty acids; TG: triacylglycerols; *hmgcr*: 3-hydroxy-3-methylglutaryl-coenzyme A reductase; *sqle*: squalene monooxygenase; *lss*: lanosterol synthase; *msmo1*: methylsterol monooxygenase 1; *cyp7a1*: cholesterol 7 alpha-hydroxylase; Acox1/2: acyl-CoA oxidase 1/2; *acat1/2*: acetyl-CoA acetyltransferase 1/2.

In mammals, it was reported that increased FAO activity in the liver is an efficient adaptive metabolism to prevent lipid accumulation and liver disease progression [44]. The process of FAO occurs in peroxisomes and mitochondria [45–47], both of which cooperate intimately [48]. For example, mitochondrial FAO was stimulated to alleviate FFA accumulation as a compensatory mechanism when peroxisomal FAO is suppressed [49]. On the contrary, stimulation of peroxisomal FAO inhibited mitochondrial FAO by promoting malonyl-CoA formation in the liver of mice fed an HFD [50]. In the present study, 4-week HFD feeding increased Acox1 and Acox2 (peroxisomal FAO rate-limiting enzymes) but inhibited Cpt1a (mitochondrial FAO rate-limiting enzyme) protein expressions in fish liver. This result suggests a similar cooperative relationship between mitochondrial and peroxisomal FAO in fish. Notably, substrate specificity studies in mammals uncovered that ACOX1 is involved in the oxidation of straight-chain fatty acids, but ACOX2 is responsible for the degradation of the branched-chain fatty acids and uniquely plays an important role in the transformation of cholesterol into bile acid [51]. In fact, it was estimated that the proportion of peroxisomal FAO is about 30% of total FAO under normal conditions in mice. However, this proportion elevated significantly in mice under the diabetic condition as liver peroxisomal FAO was stimulated in diabetes. Therefore, it was demonstrated that peroxisomal FAO plays an important role in regulating cholesterol synthesis, and enhanced peroxisomal FAO contributed to high cholesterol level in diabetes mice [12]. Also, in the present study, the stimulated peroxisomal FAO might primarily contribute to the enhanced cholesterol and bile acid synthesis in the liver of fish fed a short-term HFD, thereby reducing FFA accumulation and alleviating liver injury caused by lipotoxicity. Since, long-chain fatty acids as endogenous ligands for PPAR*α*, elevated hepatic FFA level in diabetic mice activated PPAR*α* and resulted in promoting the transcription of genes related to peroxisomal FAO [52, 53]. Our previous study in Nile tilapia also indicated that the activation of PPAR*α* by fenofibrate significantly promoted mitochondrial and peroxisomal FAO in the liver [54]. In the present study, 4-week HFD feeding induced *pparα* gene expression, which possibly contributed to the enhanced peroxisomal FAO. These results indicated that short-term HFD feeding enhanced peroxisomal FAO-mediated cholesterol and bile acid synthesis (see Figure 5). Therefore, peroxisomal FAO may also play a critical role in regulating cholesterol and bile acid synthesis in fish.

### 4.4. Long-Term HFD Intake Suppressed Conversion of FFA to Cholesterol

Animals possess an adaptive metabolism during the short-term HFD intake. The enlargement of adipocytes, which contain many lipid droplets, is the normal physiological consequence and protective strategy against lipotoxicity from high FFA [22, 55]. However, the size or numbers of adipocytes would not continuously increase with the duration of HFD intake; thereby, the maintenance of lipid homeostasis would be disrupted after long-term HFD intake. This would result in pathological consequences such as excessive lipid deposition in other tissues such as the liver, accompanied by high levels of oxidative and inflammatory factors in animals, including fish [1, 56]. The increased reactive oxygen species (ROS) produced by lipid peroxidation in hepatocytes would seriously impair lipid metabolism and further increase lipid accumulation and cause liver damage [57]. In the present study, 8-week HFD intake further elevated serum TG and TC levels and caused higher serum ALT and AST enzyme activities, indicating impaired liver in fish after long-term HFD intake. Also, more severe lipid accumulation was observed in fish liver, as is evident by obviously accumulated TG (1.4-fold increase) and FFA (1.7-fold increase). Consistently, elevated MDA content and diminished SOD activity were observed in the liver of fish fed 8-week HFD in the present study. These results might be partially due to perturbed lipid metabolism, as HFD stimulated lipolysis and suppressed peroxisomal *β*-oxidation (Acox2) protein expression in fish liver, although it did not affect the fatty acid synthesis, autophagy, and mitochondrial *β*-oxidation. Additionally, the inhibited cholesterol synthesis might link to deficient peroxisomal FAO, which provided substrate acetyl-CoA in fish liver. The disturbance of cholesterol metabolism caused by HFD intake is a critical factor for FC accumulation [2, 58]. Previous study showed that rats with HFD-induced obesity presented cholesterol accumulation in the liver via inhibiting CYP7A1 gene expression, which remarkably decreased the conversion of cholesterol to bile acids [58]. In Nile tilapia, long-term HFD feeding caused hepatic cholesterol accumulation mainly due to impaired assembly of VLDL and HLD particles [2]. However, in the present study, 8-week HFD feeding significantly elevated liver FC content that might be associated with compromised esterification of cholesterol. Additionally, LDL-C content and its receptor *ldlr* gene expression were increased, whereas cholesterol efflux and bile acid synthesis did not change in the liver of fish fed HFD. Accordingly, declined serum LDL-C level was observed in fish after 8-week HFD intake, accompanied by unaltered TBA levels in the liver and intestinal content. These results suggested that accumulated hepatic cholesterol resulted from the increased LDL-C uptake from the blood, and long-term HFD intake also caused the disturbance of cholesterol metabolism in fish. The contradictory conclusions from various studies might be due to the discrepancy between species and their nutrition status. Of note, the remarkably elevated FFA content (about 1.7-fold increase) was found in the liver of fish, along with suppressed Acox2 protein level and downregulated cholesterol/bile acid synthesis. Hence, the deficient liver peroxisomal FAO might block the conversion of FFA to cholesterol and bile acids, thereby failing to alleviate FFA accumulation in fish after 8-week HFD intake. Therefore, the elevated FFA and FC contents primarily accounted for liver lipotoxicity and injury in fish fed a long-term HFD (see Figure 5).

## 5. Conclusion

The present study revealed that short-term HFD intake triggered protective mechanisms against FFA accumulation and liver injury. Of note, short-term HFD feeding caused cholesterol accumulation (mainly CE) rather than TG in fish liver, along with increased TBA content in liver and intestinal content. Furthermore, the stimulated hepatic peroxisomal FAO, which provides substrate acetyl-CoA for the biosynthesis of cholesterol, might primarily contribute to cholesterol and bile acid synthesis and efflux, thereby alleviating FFA accumulation in the liver of fish during the short-term HFD intake. However, long-term HFD intake caused evident elevation of TG and FFA, along with accumulated FC content in fish liver. The elevated FC content resulted from the enhancement of LDL-C uptake indicating long-term HFD intake also caused the disturbance of cholesterol metabolism. Of note, long-term HFD intake suppressed peroxisomal FAO and cholesterol/bile acid synthesis, indicating the blocked conversion of FFA to cholesterol and bile acids, thereby failing to alleviate FFA accumulation in fish liver. Therefore, the robust cholesterol-bile acid flux serves as an adaptive metabolism in Nile tilapia fed an HFD, possibly via stimulating peroxisomal FAO pathway. This finding enlightens our understanding of the adaptive characteristics of cholesterol metabolism in fish fed an HFD and provides a new possible treatment strategy against metabolic disease induced by HFD in aquatic animals.

## Figures and Tables

**Table 1:** The formulation and proximate analysis of experimental diets.

Ingredients (g/kg)	Control	HFD
Casein	320.00	320.00
Gelatin	90.00	90.00
Corn starch	320.00	320.00
Soybean oil	60.00	130.00
Vitamin^1^	15.00	15.00
Minerals^2^	25.00	25.00
CMC	24.75	24.75
Cellulose	130.00	60.00
Choline chloride	5.00	5.00
Dimethyl-*β*-propiothetin	0.25	0.25
Ca(H_2_PO_4_)_2_	10.00	10.00
Total	1000	1000
Proximate composition (g kg^−1^ dry matter)		
Crude protein	393.00	388.00
Crude lipid	63.00	133.00
Ash	58.00	61.00

^1^Vitamin premix (mg or IU/kg): 500,000 I.U. (international units) vitamin A; 50,000 I.U. vitamin D3; 2500 mg vitamin E; 1000 mg vitamin K3; 5000 mg vitamin B1; 5000 mg vitamin B2; 5000 mg vitamin B6; 5000 *μ*g vitamin B12; 25,000 mg inositol; 10,000 mg pantothenic acid; 100,000 mg choline; 25,000 mg niacin; 1000 mg folic acid; 250 mg biotin; 10,000 mg vitamin C. ^2^Mineral premix, (g/kg): 314.0 g CaCO_3_; 469.3 KH_2_PO_4_; 147.4 g MgSO_4_·7H_2_O; 49.8 g NaCl; 10.9 g Fe(II) gluconate; 3.12 g MnSO_4_·H_2_O; 4.67 g ZnSO_4_·7H_2_O; 0.62 g CuSO_4_·5H_2_O; 0.16 g; 0.08 g CoCl_2_·6H_2_O; 0.06 g NH_4_ molybdate; 0.02 g NaSeO_3_.

**Table 2:** The primer sequences used in the experiment.

Gene	Accession no.	Forward primer (5′- to 3′-)	Reverse primer (5′- to 3′-)
*hmgcr*	XM_003437610.5	TGGTTAGGTCACGATGCCAC	GGGCCGGATAACCTCATCTTT
*sqle*	XM_003453510.5	GCCTGTCTTTGTTACCTGTT	GCCCGTTTCCACTCTGTTC
*msmo1*	XM_003449688.4	AGTGCTTCGGCTGTGCT	ATGGTGGACTTTGTGGATGT
*lss*	XM_025903774.1	CACAGACGCTCGGAGTT	AGATGAATAAGGGAGGTTTC
*acat1* *acat2*	XM_005479143.4 XM_003451859.5	TGATTCGGTGGGGCTATGTG GCATGATTTTGGGGTGCCTG	ACCATGGCTCGCAGATCAAA GGTCTGGGTTCTGAAGACCG
*cyp7a1*	XM_003456729.5	GGGATAAGACACAGGCAACCA	TGCGGAGGAATTGAAGTGGG
*ldlr*	XM_003443172.5	CACTGCAAGGCCATTGGAAC	GGCGAGGGATTACTCTCGTG
*abca1*	XM_005454754.4	ACACCAGACACTCCAGCAA	ACCTCCTCCCACATCCC
*abcg1*	XM_005462721.3	GGGGATGAAGGGCGAGAT	TGGACACTGAGGTGAGGC
*abcg5*	XM_003438434.5	ACGAGTCCTTCAGGTTTGTGG	TTTCCTGAGCCTGAGTTGCC
*abcg8*	XM_019366916.2	GTCGGGATGAAGGAGGC	TCGGACGGTGAGGTGAG
*fasn*	GU433188	TCATCCAGCAGTTCACTGGCATT	TGATTAGGTCCACGGCCACA
*acly*	XM_003442027	AAAAGCTTTGATGAGCTTGGGG	TACAGTGGGAGGAGGCAACTCTT
*acca*	XM_005471970	TAGCTGAAGAGGAGGGTGCAAGA	AACCTCTGGATTGGCTTGAACA
*atgl*	XM_003440346.5	GACACATGCTGCAAAGCACT	ACCAGGACGTTTTCTCCGTC
*hsl*	XM_005463937.4	AGTTCACTCCAGCCATTCGG	TGGCTGCTACCCCTATTCCT
*mgl*	XM_005478351.4	GGGCTCCATCGAGTCCAAAT	AATGATACTCGCATCCCGCC
*atg5*	XM_003450274.5	ACAGCGTCTTACCCTGGAGCA	TCACAGAGCTGGATGGGCAGT
*atg7*	XM_003454570.5	TCTCTCAGACCACTCTGTCCC	AGCAGCATTCACCACTAGCTT
*beclin*	XM_005471281.3	ACCATCAACAACTTCCGCCT	TCTGAAAGTGCAGCCCCATT
*srebp1*	XM_005471970	TGCAGCAGAGAGACTGTATCCGA	ACTGCCCTGAATGTGTTCAGACA
*pparα*	KF871430	GTTCCTCAAGAGTCTCCGCC	AAAGAGCTAGGTCGCTGTCG
*cpt1a*	XM_003440552	TTTCCAGGCCTCCTTACCCA	TTGTACTGCTCATTGTCCAGCAGA
*β-actin*	KJ126772.1	AGCCTTCCTTCCTTGGTATGGAAT	TGTTGGCGTACAGGTCCTTACG
*ef1a*	KJ123689.1	ATCAAGAAGATCGGCTACAACCCT	ATCCCTTGAACCAGCTCATCTTGT

Note: *hmgcr*: 3-hydroxy-3-methylglutaryl-coenzyme A reductase; *sqle*: squalene monooxygenase; *lss*: lanosterol synthase; *msmo1*: methylsterol monooxygenase 1; *acat1*: acetyl-CoA acetyltransferase 1; *acat2*: acetyl-CoA acetyltransferase 2; *cyp7a1*: cholesterol 7alpha-hydroxylase; *ldlr*: low-density lipoprotein receptor; *abca1*: ATP binding cassette subfamily A member 1; *abcg1*: ATP binding cassette subfamily G member 1; *abcg5*: ATP binding cassette subfamily G member 5; *abcg8*: ATP binding cassette subfamily G member 8; *fasn*: fatty acid synthase; *acly*: ATP citrate lyase; *accα*: acetyl-CoA carboxylase alpha; *atgl*: adipose triglyceride lipase; *hsl*: hormone-sensitive lipase; *mgl*: monoacylglycerol lipase; *atg5*: autophagy-related gene 5; *atg7*: autophagy-related gene 7; *srebp1*: sterol regulatory element binding protein 1; *pparα*: peroxisome proliferator-activated receptor *α*; *cpt1a*: carnitine palmitoyl transferase 1a.

## Data Availability

The datasets generated during the current study are available from the corresponding author on reasonable request.
